# Lipopolysaccharide pretreatment increases protease-activated receptor-2 expression and monocyte chemoattractant protein-1 secretion in vascular endothelial cells

**DOI:** 10.1186/s12929-017-0393-1

**Published:** 2017-11-15

**Authors:** Hung-Hsing Chao, Po-Yuan Chen, Wen-Rui Hao, Wei-Ping Chiang, Tzu-Hurng Cheng, Shih-Hurng Loh, Yuk-Man Leung, Ju-Chi Liu, Jin-Jer Chen, Li-Chin Sung

**Affiliations:** 1 Division of Cardiovascular Surgery, Department of Surgery, Shin Kong Wu Ho-Su Memorial Hospital, Taipei, 111 Taiwan; 2 Department of Surgery, School of Medicine, Taipei Medical University, Taipei, 11031 Taiwan; 3 Department of Biological Science and Technology, College of Biopharmaceutical and Food Sciences, China Medical University, Taichung, 40402 Taiwan; 4 Division of Cardiology, Department of Internal Medicine, Shuang Ho Hospital, Taipei Medical University, No. 291, Zhongzheng Rd, Zhonghe District New Taipei City, 23561 Taiwan; 5 Department of Biochemistry, School of Medicine, China Medical University, Taichung, 40402 Taiwan; 6 Department of Pharmacology & Graduate Institute of Pharmacology, National Defense Medical Center, Taipei, 114 Taiwan; 7 Department of Physiology, School of Medicine, China Medical University, Taichung, 40402 Taiwan; 8 Department of Internal Medicine, School of Medicine, College of Medicine, Taipei Medical University, Taipei, 11031 Taiwan; 9 Graduate Institute of Clinical Medicine, College of Medicine, China Medical University, Taichung, 40402 Taiwan; 10 Institute of Biomedical Sciences, Academia Sinica, Taipei, 115 Taiwan

**Keywords:** Lipopolysaccharides, Protease-activated receptor-2, Endothelial cells, Monocyte chemoattractant protein-1, Mitogen-activated protein kinases

## Abstract

**Background:**

This study investigated whether lipopolysaccharide (LPS) increase protease-activated receptor-2 (PAR-2) expression and enhance the association between PAR-2 expression and chemokine production in human vascular endothelial cells (ECs).

**Methods:**

The morphology of ECs was observed through microphotography in cultured human umbilical vein ECs (EA. hy926 cells) treated with various LPS concentrations (0, 0.25, 0.5, 1, and 2 μg/mL) for 24 h, and cell viability was assessed using the MTT assay. Intracellular calcium imaging was performed to assess agonist (trypsin)-induced PAR-2 activity. Western blotting was used to explore the LPS-mediated signal transduction pathway and the expression of PAR-2 and adhesion molecule monocyte chemoattractant protein-1 (MCP-1) in ECs.

**Results:**

Trypsin stimulation increased intracellular calcium release in ECs. The calcium influx was augmented in cells pretreated with a high LPS concentration (1 μg/mL). After 24 h treatment of LPS, no changes in ECs viability or morphology were observed. Western blotting revealed that LPS increased PAR-2 expression and enhanced trypsin-induced extracellular signal-regulated kinase (ERK)/p38 phosphorylation and MCP-1 secretion. However, pretreatment with selective ERK (PD98059), p38 mitogen-activated protein kinase (MAPK) (SB203580) inhibitors, and the selective PAR-2 antagonist (FSLLRY-NH2) blocked the effects of LPS-activated PAR-2 on MCP-1 secretion.

**Conclusions:**

Our findings provide the first evidence that the bacterial endotoxin LPS potentiates calcium mobilization and ERK/p38 MAPK pathway activation and leads to the secretion of the pro-inflammatory chemokine MCP-1 by inducing PAR-2 expression and its associated activity in vascular ECs. Therefore, PAR-2 exerts vascular inflammatory effects and plays an important role in bacterial infection-induced pathological responses.

**Electronic supplementary material:**

The online version of this article (10.1186/s12929-017-0393-1) contains supplementary material, which is available to authorized users.

## Background

The important role of bacterial endotoxins in the pathophysiology of sepsis was recognized in the 1960s and 1970s [1]. Lipopolysaccharides (LPS), also called endotoxins, are expressed by most gram-negative bacteria and play an important role in the function and structural integrity of the outer lipid membrane [2]. LPSs are a family of large molecules containing three structural elements: a core oligosaccharide, an O-antigen, and a lipid A component [2–4]. High LPS levels have certain toxic effects on cells, whereas low LPS levels promote cell proliferation. Epidemiological studies have indicated that LPS constitute a risk factor for diseases such as atherosclerosis and diabetes [4].

The endothelium also plays a major role in the pathogenesis of sepsis. Endothelial cells (ECs) line the inner wall of blood vessels, lying at the interface between circulating blood and the surrounding tissue [3]. During infection, LPS bind to the surface of ECs, resulting in the activation of endothelial signaling pathways and the release of inflammatory mediators [3, 5, 6]. These mediators induce the production of reactive oxygen species, secretion of chemokines and adhesion molecules, reduction of anti-inflammatory mediators, and transmigration of leukocytes [5, 7, 8]. The infection-induced inflammatory reaction is further mediated by complex interactions between circulating leukocytes and the vascular endothelium [3, 7, 9, 10]. The adherence of monocytes to the activated endothelium and their subsequent proliferation are critical for atherosclerotic plaque formation [7, 11–15]. Chemokines produced by ECs are vital for promoting the movement of circulating monocytes to atherosclerotic vessels and the infection site [9, 14, 16]. Monocyte chemoattractant protein-1 (MCP-1), a potent chemoattractant for monocytes, is closely involved in atherosclerosis development [5, 11, 12, 17, 18]. Studies have observed elevated plasma MCP-1 levels in patients with coronary artery disease, with the highest levels being observed in those with acute coronary syndrome and diabetes [17, 19, 20]. Although LPS-induced MCP-1 secretion from the vascular endothelium has been reported to recruit circulating monocytes, the underlying mechanism remains largely unexplained [9, 21].

Protease-activated receptor-2 (PAR-2) is a member of the G protein-coupled receptor family with seven transmembrane-spanning domains, and it is mainly activated by trypsin [22–25]. PAR-2 is a key mediator of innate immunity and inflammatory response propagation [26]. Endothelial PAR-2 is mainly activated by the locally released trypsin that accompanies tissue injury or inflammation. PAR-2 is widely expressed in nearly all cell types in the vascular wall (ECs, myocytes, and fibroblasts) [27–29]. Several studies have revealed that PAR-2 is involved in inflammation and endotoxin shock [24, 27]. The expression of PAR-2 was increased 5- to 10-fold in ECs after LPS exposure in vitro, thus suggesting the possible involvement of PAR-2 in endotoxemia [30]. Immunohistochemical studies have demonstrated preferential and localized increases in the expression of PAR-2 in the aorta and jugular vein, and these increases were associated with endotoxin shock [27, 30]. Furthermore, enhanced PAR-2 expression has been observed in human coronary atherosclerotic lesions, suggesting that PAR-2 regulates signaling during vascular injures [27, 31]. Increasing bodies of evidence from cellular and animal studies reveal that PAR-2 activation is associated with increased MCP-1 secretion [32–34]. However, the relationship between the PAR-2 signaling pathway and LPS-activated MCP-1 secretion remains unclear [35].

The present study investigated whether LPS activates PAR-2 expression and consequently enhances trypsin-induced PAR-2 signaling and subsequent MCP-1 secretion in human vascular ECs.

## Methods

### Materials

Unless stated otherwise, trypsin, purified LPS (obtained through phenol extraction) from *Escherichia coli* (serotype O26:B6), salts, buffers, and all other chemicals of reagent grade were purchased from Sigma-Aldrich (St. Louis, MO, USA). The specific PAR-1 agonist (TRAP6), PAR-2 agonist (AC 55541), PAR-4 agonist (AY-NH2) and the selective PAR-2 antagonist (FSLLRY-NH2) were purchased from Tocris Bioscience (Bristol, UK). Antibody-directed phosphorylated ERK was purchased from Novus (St. Charles, MO, USA), and anti-ERK was purchased from BD (Franklin Lakes, NJ, USA). Antiphosphorylated p38, anti-p38, and anti-c-JUN N-terminal kinase (JNK) were purchased from Calbiochem (San Diego, CA, USA). Anti-MCP-1 was purchased from Sigma-Aldrich. Monoclonal antiphosphorylated JNK, anti-PAR-2 (Additional file 1: Figure S1), and anti-β-actin antibodies were purchased from Santa Cruz Biotechnology (Santa Cruz, CA, USA).

### EA. hy926 cells

The human EC line, EA. hy926, was originally derived from a human umbilical vein obtained from the American Type Culture Collection (Manassas, VA, USA). The cells were grown in Dulbecco’s Modified Eagle’s Medium/Ham’s Nutrient Mixture F-12 (DMEM/F12; 1:1, Life Technologies, Grand Island, NY, USA) supplemented with 10% fetal bovine serum (FBS), 1% L-glutamine, and 1% penicillin–streptomycin in a humidified atmosphere of 5% CO_2_ at 37 °C. During cell culture, the medium was changed every 3 days until the cells reached 90% confluence. To prevent FBS-induced trypsin inactivation, all cells were incubated in a FBS-free DMEM with 1% penicillin–streptomycin solution during trypsin treatment.

### Intracellular calcium release measurement

Intracellular calcium release in ECs was assessed through microfluorimetric measurements of the cytosolic Ca^2+^ concentration by using fura-2 as described previously [36]. In brief, ECs were incubated with 5 μM fura-2 AM (Invitrogen, Carlsbad, CA, USA) for 1 h at 37 °C and subsequently washed and bathed in DMEM supplemented with 10% FBS and penicillin–streptomycin solution (100 units/mL, 100 μg/mL; Invitrogen) under 5% CO_2_. The cells were alternately excited at 340 and 380 nm using an optical filter changer (Lambda 10-2, Sutter Instruments, Novato, CA, USA). Emission was measured at 500 nm, and images were captured using a charge-coupled device camera (CoolSnap HQ2, Photometrics) attached to an inverted Nikon TE 2000-U microscope. The captured images were analyzed using MAG Biosystems Software. All experiments were performed at room temperature (approximately 25 °C).

### Reverse transcription polymerase chain reaction

Total RNA was isolated using RNAzol solution (Biogenesis, Poole, Dorset, UK), according to the manufacturer’s instructions. RNA purity was estimated though optical density measurements at 260/280 nm. The derived total RNA (5 μg) was subjected to first-strand cDNA synthesis in a 10-μL reaction volume containing 250 mM Tris-HCl (pH 8.3 at 20 °C), 375 mM KCl, 15 mM MgCl_2_, 1 mM 1,4-dithiothreitol (DTT), 1 mM of each dNTP, and 20 U of an RNase inhibitor in the presence of 1.5 μg of an oligo dT primer and 200 U of Superscriptase (all chemicals were obtained from Life Technologies). After the completion of the first-strand cDNA synthesis process, the reaction was terminated by heat inactivation (5 min, 95 °C) and the derived total RNA was diluted with water to obtain 50 ng/μL of RNA equivalent. cDNA equivalent to 100 ng of the total RNA was subjected to polymerase chain reaction (PCR) in a 50-μL reaction volume, containing 10 mM Tris-HCl (pH 9 at 25 °C), 50 M KCl, 1.5 mM MgCl_2_, 0.01% (*w*/*v*) gelatin, 0.1% (*v*/v) Triton X-100, 2 mM DTT, 200 μM of each dNTP, 1 μM of each primer, and 0.2 U of TaqDNA polymerase (AB Biotechnology) under the following conditions: denaturation, 30 s at 94 °C; primer annealing, 1 min at 58 °C; and primer extension, 1 min at 72 °C. The PCR products (10 μL) were electrophoresed in 1% agarose gels and visualized through ultraviolet illumination. The PAR-2 forward and reverse primers were 5′-TGGCACCATCCAAGGAAC-3′ and 5′-GTCAGCCAAGGCCAGATT-3′, respectively. The glyceraldehyde 3-phosphate dehydrogenase (GAPDH) forward and reverse primers were 5′-ACCACAGTCCATGCCATCAC-3′ and 5′-TCCACCACCCTGTTGCTGTA-3′, respectively.

### Western blotting

Cells were lysed in a buffer containing 50 mM Tris (pH 8.0), 5 mM ethylenediaminetetraacetic acid, 5 mM ethylene glycol tetraacetic acid, 0.2% sodium dodecyl sulfate (SDS), 0.5% Nonidet P-40, 1 mM sodium orthovanadate, 20 mM sodium pyrophosphate, and Roche complete protease inhibitor mixture (Roche, Mannheim, Germany). The protein in the medium was precipitated with 10% trichloroacetic acid and 0.1% sodium deoxycholate. The precipitates were redissolved in the SDS sample buffer. The extracted protein was separated through SDS-polyacrylamide gel electrophoresis and transferred onto a polyvinylidene fluoride membrane (Bio-Rad Laboratories, Hercules, CA, USA). The membrane was blocked with 2%–3% skim milk in Tris-buffered saline (TBS) containing 0.05% Tween20; subsequently, the membrane was probed with the anti-PAR-2, anti-phospho-ERK (p-ERK), anti-total ERK or anti-phospho-p38 MAPK (p-p38), anti-total p38 MAPK, anti-phospho-JNK (p-JNK), anti-total JNK, anti-MCP-1, and anti-β-actin antibodies. After overnight incubation with different antibodies at 4 °C, the membrane was washed three times with TBS and incubated with horseradish peroxidase*-*conjugated secondary antibodies for 1 h at room temperature. Finally, the immunoblots were quantified using Image J densitometry analysis software (National Institutes of Health, Bethesda, MD, USA).

### Cell adhesion assay

EA. hy926 monolayers, grown as described previously, were established in culture dishes and subsequently treated with LPS (0, 0.25, 0.5, 1, and 2 μg/mL). After a 24 h of incubation, the EA. hy926 cells in each well were treated with trypsin (5 μg/mL) and cultured for 12 h, followed by incubation with 2 × 10^5^ peripheral blood mononuclear cells for 30 min in a humidified atmosphere of 5% CO_2_ at 37 °C [37]. After incubation, non-adherent cells were removed by washing two times with PBS. Six random high-power microscopic fields (100×) were photographed, and the number of adhered cells was directly calculated.

### Statistical analysis

All experiments were performed at least in triplicate. Data are presented as mean ± standard error of the mean (SEM). Statistical analysis was performed using the Student *t* test or analysis of variance, followed by the Dunnett multiple comparison test by using Prism software (version 3.00 for Windows GraphPad, San Diego, CA, USA). A *P* value of <0.05 was considered statistically significant.

## Results

### Analysis of PAR-2 expression in LPS-treated ECs

**Fig. 1:**
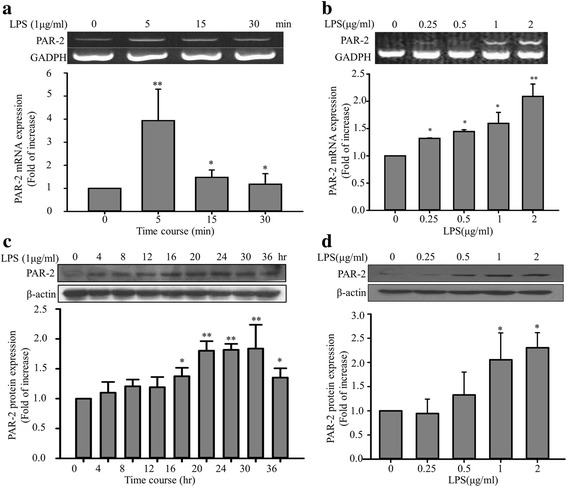
Effects of LPS pretreatment on PAR-2 expression in EA. hy926 cells. EA. hy926 cells were treated with LPS at different concentrations and treatment durations. Cellular RNA was isolated, and RT-PCR and Western blotting were performed to assess PAR-2 mRNA and protein levels, respectively. **a** EA. hy926 cells were stimulated with LPS (1 μg/mL) for 0, 5, 15, and 30 min. PAR-2 and GAPDH mRNA levels were determined through RT-PCR. The PAR-2 mRNA levels were normalized to the control values of GAPDH (%). **b** EA. hy926 cells were stimulated with different LPS concentrations (0, 0.25, 0.5, 1, and 2 μg/mL) for 15 min. **c** Effects of LPS pretreatment on PAR-2 protein expression. EA. hy926 cells were stimulated with LPS (1 μg/mL) for 0, 4, 8, 12, 16, 20, 24, 30, and 36 h. The PAR-2 protein levels were normalized to the control values of β-actin (%). **d** EA. hy926 cells were stimulated with different LPS concentrations (0, 0.25, 0.5, 1, and 2 μg/mL) for 24 h. Bar graphs represent means ± SEM from three independent experiments. *p < 0.05 compared with the control group; **p < 0.01 compared with the control group

PAR-2 is highly expressed in ECs and plays an important role in inflammation [28]. In this study, PAR-2 expression after LPS treatment was examined in vitro. Reverse transcription (RT)-PCR and Western blotting revealed increased PAR-2 mRNA and protein expression levels in EA. hy926 cells (Fig. 1). Notably, LPS increased PAR-2 expression in EA. hy926 cells. The PAR-2 mRNA levels in EA. hy926 cells were significantly elevated after LPS treatment (1 μg/mL, 5 min; *P* < 0.01 compared with the control group; Fig. 1a and b). However, the stimulating effects of LPS pretreatment on PAR-2 protein levels were not apparent after short-term LPS treatment (<12 h). By contrast, 1 μg/mL of LPS resulted in increased PAR-2 protein levels, with a peak at 20 h of treatment (Fig. 1c and d).

### LPS pretreatment enhances trypsin-induced intracellular calcium release in ECs

**Fig. 2:**
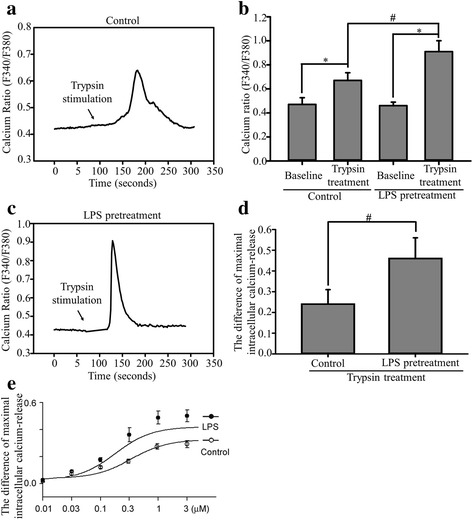
Effects of LPS pretreatment on trypsin-induced intracellular Ca^2+^ release in ECs. EA. hy926 cells were left untreated (control group) or pretreated with 1 μg/mL of LPS for 24 h (LPS pretreatment group) and stimulated with 2 μg/mL of trypsin. **a** Trypsin exposure induced an increase in intracellular Ca^2+^ release (F340/F380 nm) in EA. hy926 cells. The arrow indicates trypsin addition to the superfusion solution. **b** Bar graph of the maximal calcium release before and after trypsin stimulation in ECs. The basal intracellular Ca^2+^ release without trypsin was used as the baseline. Trypsin treatment significantly increased the maximal intracellular Ca^2+^ release. **c** Trypsin induced an increase in intracellular Ca^2+^ release in the LPS-pretreatment group. **d** Bar graph of the differences in the trypsin-induced maximal intracellular Ca^2+^ release in the control and LPS pretreatment groups. Bar graphs represent means ± SEM from six independent experiments. **e** Concentration–response curves for the trypsin-induced increase in intracellular Ca^2+^ release in the absence and presence of LPS pretreatment for 24 h in ECs. The data are presented as mean ± SEM (*n* = 6). *p < 0.05 compared with the control group; ^#^ *p* < 0.05 compared with the LPS pretreatment group

Trypsin is an endogenous PAR-2 activator [38]. We examined the effects of LPS pretreatment on trypsin-induced intracellular calcium release in ECs. The relative changes in intracellular calcium release were determined using the fura-2 F340/F380 nm ratio. Basal intracellular calcium release (prior to trypsin exposure) did not differ between the control and LPS-pretreatment groups. In addition, treatment with LPS alone (1 μg/mL, 24 h) did not alter EC viability (data not shown). In the absence of LPS pretreatment, trypsin (2 μg/mL) induced a rapid and transient increase in intracellular Ca^2+^ release in ECs (Fig. 2a). However, after LPS pretreatment (1 μg/mL, 24 h), the trypsin-induced intracellular Ca^2+^ release increased to 40% (Fig. 2c). Figure 2b presents a typical example of the averaged maximal intracellular Ca^2+^ levels induced by trypsin in the absence or presence of LPS pretreatment. The maximal increase in the trypsin-induced intracellular Ca^2+^ release was higher in the LPS-pretreatment group than in the control group (Fig. 2d). The concentration–response curve for the trypsin-induced Ca^2+^ release in the absence or presence of LPS pretreatment (1 μg/mL, 24 h) is shown in Fig. 2e. In the concentration-response curve for the trypsin-induced Ca^2+^ release after LPS pretreatment, a left shift with changing maximum response suggests the stimulating effects of LPS pretreatment on PAR-2.

### LPS pretreatment enhances trypsin-induced ERK/p38 phosphorylation in ECs

**Fig. 3:**
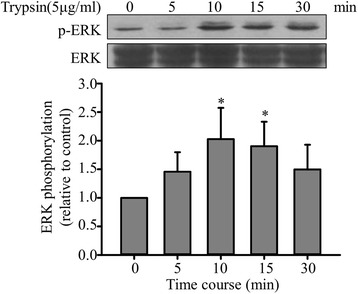
Effects of trypsin on ERK activation. Upper panel: Typical blot showing ERK phosphorylation in EA. hy926 cells treated with trypsin. EA. hy926 cells (1 × 10^6^/mL) were treated with 5 μg/mL of trypsin for the indicated duration, and p-ERK levels were determined through Western blotting as described and normalized to the total ERK levels. Lower panel: Normalization of the p-ERK and total ERK levels. Bar graphs represent means ± SEM from four independent experiments. *p < 0.05 compared with the control group

**Fig. 4:**
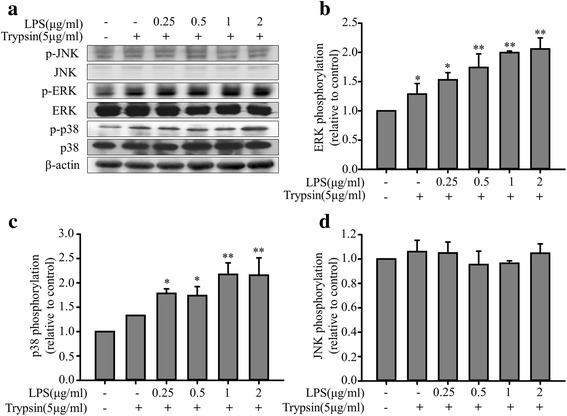
Enhancement effects of LPS pretreatment on MAPK phosphorylation in EA. hy926 cells treated with trypsin. EA. hy926 cells were treated in the absence or presence of LPS (0.25, 0.5, 1, and 2 μg/mL) for 24 h and with 5 μg/mL of trypsin for 10 min. **a** Representative data of the p-ERK, total ERK, p38, and JNK MAPK levels. **b** Normalization of the p-ERK and total ERK levels. **c** Normalization of the p-p38 and total p38 levels. **d** Normalization of the p-JNK and total JNK levels. Bar graphs represent means ± SEM from four independent experiments. *p < 0.05 compared with the control group; **p < 0.01 compared with the control group

A study demonstrated that PAR-2 activation can influence cellular functions through several signal transduction pathways [25]. We thus investigated the trypsin-induced phosphorylation of ERK and observed that p-ERK levels were significantly higher in EA. hy926 cells stimulated with trypsin (5 μg/mL, 10 min) than in untreated cells (Fig. 3). Subsequently, the observed ERK phosphorylation subsided gradually. However, after a 24 h of LPS pretreatment, trypsin enhanced the phosphorylation of ERK and p38 (Fig. 4a). The p-ERK and p-p38 levels were significantly enhanced in the LPS-pretreatment group supplemented with trypsin (Fig. 4b and c). However, compared with trypsin alone, the combined treatment of trypsin and LPS did not significantly enhance JNK phosphorylation (Fig. 4d).

### LPS pretreatment enhances trypsin-induced MCP-1 secretion and cell adhesion in ECs

**Fig. 5:**
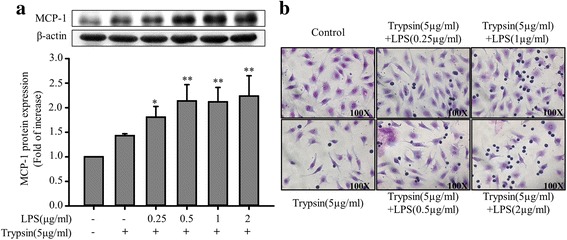
Enhancement effects of LPS pretreatment on MCP-1 secretion and cell adhesion functions in EA. hy926 cells treated with trypsin. EA. hy926 cells were treated in the absence or presence of LPS (0.25, 0.5, 1, and 2 μg/mL) for 24 h, followed by treatment with 5 μg/mL of trypsin for 12 h. **a** MCP-1 protein levels were quantified through immunoblotting, and β-actin was used as the loading control. The upper panel shows a typical blot. Bar graphs represent means ± SEM from four independent experiments. *p < 0.05 compared with the control group; **p < 0.01 compared with the control group **b** Effects of LPS pretreatment on trypsin-induced mononuclear cell–EC adhesion. ECs were treated with the indicated LPS concentrations (0, 0.25, 0.5, 1, and 2 μg/mL) for 24 h, followed by treatment with trypsin (5 μg/mL) for 12 h. The adhesion assay with mononuclear cells was performed in sextuplicate. Microphotographs (100×) show the adhesion of mononuclear cells to the trypsin-stimulated ECs treated with the indicated LPS concentrations

**Fig. 6:**
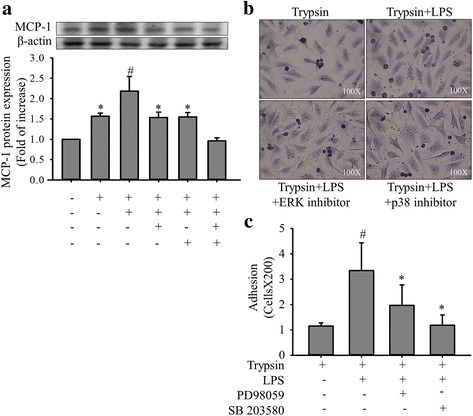
Inhibitory effects of PD98059 and SB203580 on MCP-1 secretion and cell adhesion functions in EA. hy926 cells. EA. hy926 cells (1 × 10^6^/mL) were pretreated with PD98059 (25 μM), SB203580 (10 μM), or PD98059 (25 μM) and SB203580 (10 μM) for 30 min, after which they were stimulated with LPS (1 μg/mL) for 24 h and with trypsin (5 μg/mL) for 12 h. Control cells were treated with 0.1% DMSO. **a** MCP-1 protein levels were quantified through immunoblotting, and β-actin was used as the loading control. The upper panel shows a typical blot. Bar graphs represent means ± SEM from four independent experiments. ^#^p < 0.05 compared with the trypsin treatment only group; *p < 0.05 compared with the trypsin plus LPS-pretreatment group; **p < 0.01 compared with the trypsin plus LPS-pretreatment group **b** Effects of PD98059 and SB203580 on trypsin-induced mononuclear cell–EC adhesion in LPS-pretreated ECs. Microphotographs (100×) show the adhesion of mononuclear cells to ECs treated as described previously. **c** The adhesion assay was performed using mononuclear cells. Bar graphs represent means ± SEM from four independent experiments. ^#^p < 0.05 compared with the trypsin treatment only group; *p < 0.05 compared with the trypsin plus LPS-pretreatment group

PAR-2 activation is associated with the adhesion of leukocytes to the vascular endothelium [10]. However, whether LPS pretreatment regulates trypsin-induced cell adhesion molecule expression and cell adhesion functions remains unclear. Our results revealed that compared with trypsin alone, the combined treatment of LPS (0.25, 0.5, 1, and 2 μg/mL; 24 h) and trypsin (5 μg/mL) enhanced MCP-1 protein secretion (Fig. 5a). To assess the effects of LPS pretreatment on trypsin-induced mononuclear cell adhesion, EA. hy926 cells were treated with different LPS concentrations (0, 0.25, 0.5, 1, and 2 μg/mL) for 24 h, followed by treatment with trypsin (5 μg/mL) for 12 h. Mononuclear cells were added to the EC culture to assess cell adhesion functions. When trypsin stimulation was not applied very few mononuclear cells adhered to the ECs; however, trypsin clearly increased mononuclear cell–EC adhesion. In addition, LPS pretreatment enhanced trypsin-induced mononuclear cell–EC adhesion in a concentration-dependent manner (Fig. 5b). Notably, the combined treatment of LPS and trypsin exerted more significant regulatory effects on cell adhesion functions than did trypsin alone. Furthermore, to investigate the role of trypsin-induced ERK and p38 phosphorylation, LPS-pretreated EA. hy926 cells were pre-incubated with PD98059 (25 μM), SB203580 (10 μM), or PD98059 (25 μM) and SB203580 (10 μM) for 30 min (Fig. 6a), which significantly inhibited the enhancement effects of LPS pretreatment on trypsin-induced MCP-1 secretion. Similarly, pre-incubation with PD98059 (25 μM) or SB203580 (10 μM) inhibited the promotive effects of LPS pretreatment on trypsin-induced cell adhesion functions (Fig. 6b and c). PD98059 and SB203580 also influenced the regulatory effects of LPS pretreatment on MCP-1 secretion and cell adhesion functions in trypsin-treated EA. hy926 cells.

### The relationship of PAR-2 activity and the levels of MCP-1 production in response to the combined treatment of trypsin and LPS

**Fig. 7:**
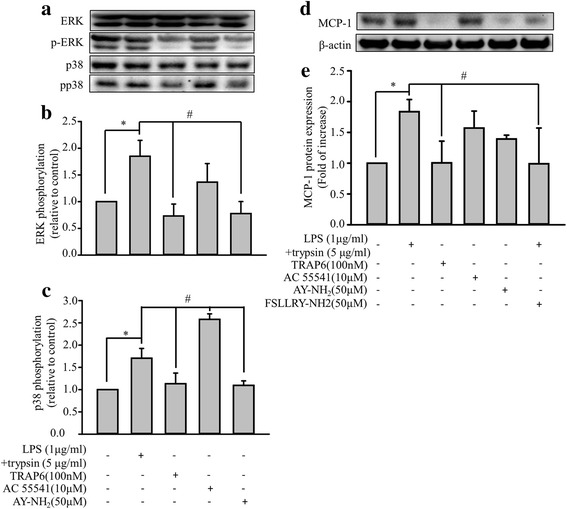
Effects of LPS-plus-trypsin, selective PAR (PAR-1, PAR-2, PAR-4) agonists, and PAR-2 antagonist in EA. hy926 cells. **a** Effects of LPS-plus-trypsin, and selective PAR (PAR-1, PAR-2, PAR-4) agonists on ERK/p38 phosphorylation. EA. hy926 cells (1 × 10^6^/mL) were treated with LPS (1 μg/mL) for 24 h and then with trypsin (5 μg/mL) for 10 min, PAR-1 agonist (TRAP6, 100 nM), PAR-2 agonist (AC 55541, 10 μM), or PAR-4 agonist (AY-NH2, 50 μM) for 24 h. Control cells were treated with 0.1% DMSO. Representative data of the p-ERK, total ERK, p38, and pp38 MAPK levels. **b** Normalization of the p-ERK and total ERK levels. **c** Normalization of the p-p38 and total p38 levels. Bar graphs represent means ± SEM from three independent experiments. *p < 0.05 compared with the control group. **d** Effects of LPS-plus-trypsin, selective PAR (PAR-1, PAR-2, PAR-4) agonists, and PAR-2 antagonist (FSLLRY-NH2 50 μM) on MCP-1 secretion. EA. hy926 cells (1 × 106/mL) were treated with LPS (1 μg/mL) for 24 h and then with trypsin (5 μg/mL) for 10 min, PAR-1 agonist (TRAP6, 100 nM), PAR-2 agonist (AC 55541, 10 μM), PAR-4 agonist (AY-NH2, 50 μM) for 24 h, or pretreated with PAR-2 antagonist (FSLLRY-NH2, 50 μM) for 30 min then LPS-plus-trypsin treatment. Control cells were treated with 0.1% DMSO. MCP-1 protein levels were quantified through immunoblotting, and β-actin was used as the loading control. The upper panel shows a typical blot. **e** Normalization of the MCP-1 and total β-actin levels. Bar graphs represent means ± SEM from three independent experiments. *p < 0.05 compared with the control group. #p < 0.05 compared with the LPS-plus-trypsin treatment group

There are four known protease-activated receptors (PAR 1-4). PAR-1, PAR-3, and PAR-4 can be activated by thrombin, and PAR-2 can be mainly activated by trypsin and numerous studies have demonstrated that PAR-2 is highly expressed in ECs [23–25, 27, 39]. To further rule out the possibility of the signaling coming from either PAR-1 or PAR-4 activation in ECs since trypsin can also activate both of these receptors, selective peptide agonists were used. PAR-1 agonist (TRAP6, 100 nM) or PAR-4 agonist (AY-NH2, 50 μM) was used to examine its effect on ERK/p38 phosphorylation and MCP-1 secretion in ECs (Fig. 7). TRAP6 failed to show increased ERK/p38 phosphorylation and MCP-1 secretion. However, the addition of AY-NH2 mildly increased MCP-1 secretion (Fig. 7d). The underlying mechanism of PAR-4 activation in the modulation of MCP-1 secretion is unknown, which should be validated in future study. In the same time, we also used a specific PAR-2 agonist (AC 55541, 10 μM) to compare with the effect of LPS plus trypsin on the stimulation of ERK/p38 phosphorylation and MCP-1 secretion in ECs. The result showed a similar pattern of stimulatory effect between PAR-2 agonist treated group and the LPS plus trypsin treated group. In addition, we also examined the effect of the selective PAR-2 antagonist (FSLLRY-NH2, 50 μM) on the induction of MCP-1 secretion by LPS plus trypsin treatment. As shown in Fig. 7d, the application of the PAR-2 antagonist specifically inhibited the induction of MCP-1 secretion by LPS plus trypsin treatment. These findings suggest that LPS plus trypsin treatment regulated the related signaling pathway mainly through PAR-2 activation.

## Discussion

**Fig. 8:**
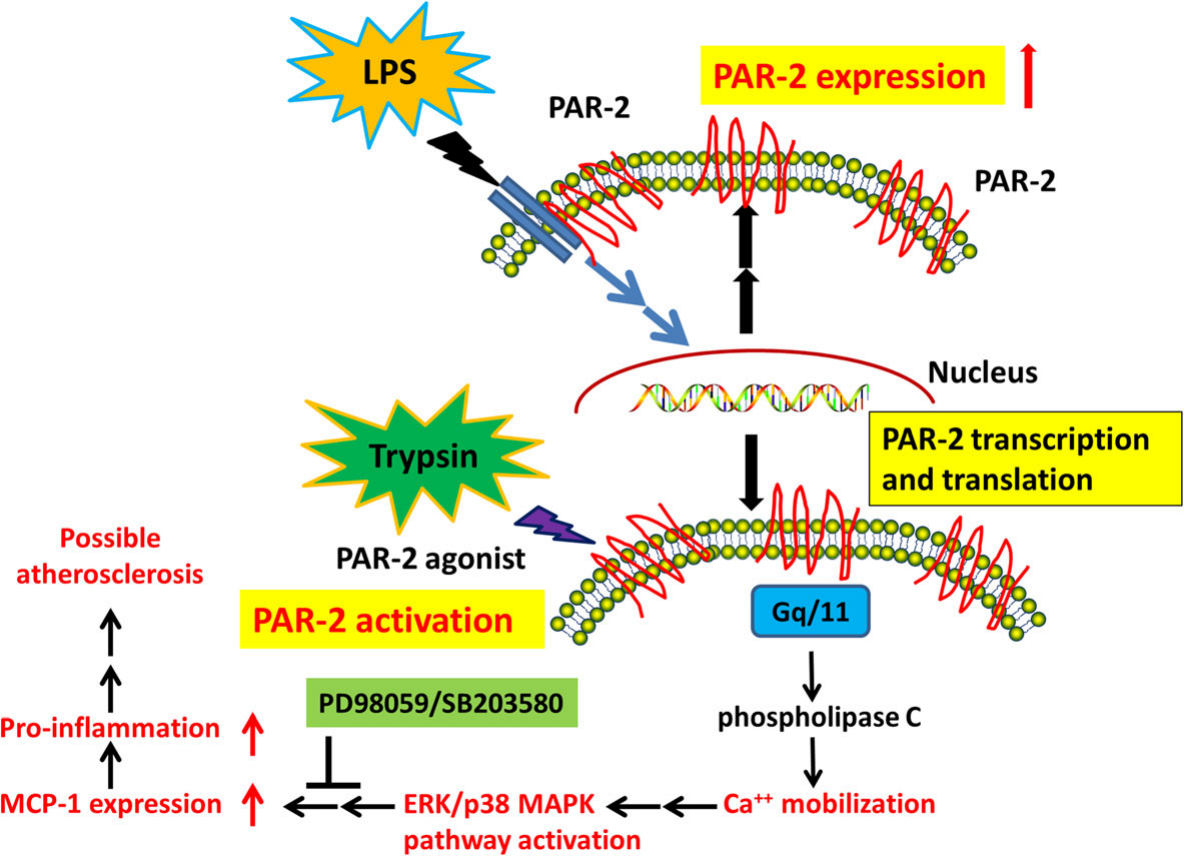
Schematic illustration of the proposed mechanism for infection-induced pathological responses. LPS pretreatment induced PAR-2 expression in vascular ECs. PAR-2 agonist (trypsin) potentiates PAR-2 activation and then calcium mobilization. The intracellular calcium activates the ERK/p38 MAPK pathway and leads to MCP-1 secretion

The present study provides the first evidence that LPS pretreatment potentiates calcium mobilization and ERK/p38 MAPK pathway activation and subsequently leads to MCP-1 secretion by inducing PAR-2 gene expression in vascular ECs. In addition, pretreatment with selective inhibitors of ERK (PD98059), p38 (SB203580), or both suppressed LPS-induced MCP-1 secretion and cell adhesion functions in ECs (Fig. 8).

According to the previous studies, LPS has potent pro-inflammatory properties, which can activate recognition receptors on ECs, leading to the release of inflammatory mediators [7, 21]. Inflammatory mediators function in autocrine and paracrine loops to further activate the monocyte and local endothelium [7]. The combined effects of LPS and inflammatory mediators on the endothelium may engender significant pathological changes. The Bruneck study provided the first epidemiological evidence that circulating LPS constitute strong risk factor for carotid atherosclerosis [5]. Moreover, LPS accelerated the development of atherosclerotic plaques in rabbits on hypercholesterolemic diets and in mice with apolipoprotein E-deficient [5]. In healthy humans, an LPS dose of 1 ng/kg is sufficient to induce symptoms including fever and nausea [40]. In a clinical observational study, the median endotoxin level in patients with sepsis was 300 pg/mL [6]. In in vitro studies on ECs, the LPS concentration range used in the basic experiments was 0.1–10 μg/mL [21, 41, 42]. Therefore, the use of different LPS concentrations in our experiment is reasonable. However, the data presented in the present in vitro study of an LPS-induced inflammatory model do not fully represent the in vivo action of LPS; hence, the results of this study warrant further validation in additional animal models.

PAR-2 was originally cloned in 1994 and plays major pathophysiological roles in angiogenesis, tissue regeneration, and inflammation [33]. Several studies have reported that PAR-2 exerts extensive effects on inflammatory responses in vascular tissues, and that LPS exposure results in increased PAR-2 levels, both in vitro and in vivo, thus suggesting the possible role of PAR-2 in endotoxemia [10, 24, 43]. We demonstrated that LPS increases PAR-2 mRNA and protein expression in ECs. Moreover, trypsin is a potent PAR-2 activator that cleaves and triggers PAR-2 activation [25]. Trypsin-induced PAR-2 activation increases intracellular calcium release through the activation of phospholipase C isoforms by using several Gq/G11-coupled receptor-modulated intracellular targets [27, 29]. Furthermore, Ca^2+^ signaling activates tyrosine kinases, which contribute to mitogen-activated protein kinase (MAPK) activation in ECs [44, 45]. Previous studies have demonstrated that PAR-2 can activate multiple kinase pathways, including the extracellular signal-regulated kinase (ERK)/p38 MAPK pathway, in a cell type-specific manner [29, 46]. In the present study, we analyzed the combined effects of LPS and trypsin on PAR-2 activation in ECs. LPS pretreatment enhances trypsin-induced intracellular Ca^2+^ release, ERK/p38 phosphorylation, and MCP-1 secretion. To elucidate the role of cytosolic calcium in the signaling pathway, we added BAPTA-AM, an intracellular calcium chelator, to identify it. The addition of BAPTA-AM slightly suppressed the LPS-induced ERK/p38 phosphorylation and significantly inhibited MCP-1 synthesis (Additional file 2: Figure S2), implying that calcium signaling is involved in the pathway. The inhibition of PAR-2 activity by a selective PAR-2 peptide antagonist (FSLLRY-NH2) also blocked the induction of MCP-1 secretion by LPS plus trypsin treatment, supporting our hypothesis that PAR-2 plays an important role in the process. Therefore, we concluded that LPS and trypsin can synergistically stimulate the PAR-2 signaling pathway.

Previous studies have demonstrated that PAR-2 activation in vascular ECs significantly increases monocyte recruitment, possibly through chemokine induction [32–34]. The transmigration of monocytes to sub-endothelial lesions is the initial step of atherosclerotic plaque formation [12, 31, 47]. MCP-1, a glycoprotein with an apparent molecular mass of 14 kDa, is produced by smooth muscle cells, ECs, and macrophages; MCP-1 is thus a highly potent chemoattractant for monocytes [48]. MCP-1 is highly expressed in human atherosclerotic plaques and is crucial in monocyte recruitment into sub-endothelial lesions [5, 18, 19]. Some studies have reported that LPS induces MCP-1 secretion from the vascular endothelium; however, the underlying mechanism is not yet clearly understood [9, 21]. According to our study results, we speculate that LPS and trypsin-activated PAR-2 can induce MCP-1 secretion.

Previous studies have showed that PAR-2 activates ERK and p38 MAPK in non-ECs [25, 29, 33, 46]. In the present study, incubation with trypsin or LPS resulted in significant ERK and p38 MAPK phosphorylation and activation in EA. hy926 cells. However, pretreatment with selective ERK and p38 MAPK inhibitors blocked the promotive effects of trypsin and/or LPS-activated PAR-2 on MCP-1 secretion. The p38 MAPK signaling pathway plays an important role in mediating pro-inflammatory responses in ECs [7, 49]. Additionally, previous studies have demonstrated that the oral administration of a specific p38 MAPK inhibitor reduces cytokine production, leukocyte responses, and inflammation in a human endotoxemia model [7, 49]. Taken together, these data suggest that PAR-2 signaling through the MAPK pathways results in increased MCP-1 secretion in ECs.

The present in vitro model of LPS-induced MCP-1 secretion through the PAR-2 signaling pathway may not be directly translatable to clinical investigations of atherosclerotic cardiovascular disease. Nevertheless, the presented preliminary results may encourage further research on identifying the molecular mechanisms underlying PAR-2-mediated MCP-1 secretion and vascular inflammation.

## Conclusions

In summary, our results reveal that PAR-2 plays an important role in regulating MCP-1 secretion through the ERK/p38 MAPK signaling pathway, thus demonstrating that PAR-2 directly modulates endothelial functions and EC–monocyte interactions by regulating MCP-1 protein release. Our findings also provide evidence that the PAR-2 signaling pathway exerts inflammatory effects on vascular ECs, leading to the initiation of infection-induced pro-atherogenic inflammatory responses. This information can be used to develop new strategies for preventing the development of atherosclerotic cardiovascular disease.

## Additional file

Additional file 1: Figure S1. Western blot – Anti-PAR-2 antibody. Lane A: Control cell lysate at 10 μg. Lane B: PAR-2 agonist (AC 55541, 10 μM)-treated cell lysate at 10 μg. Predicted band size: 43~55 kDa. (TIFF 823 kb)
Additional file 2: Figure S2. The effect of calcium chelator (BAPTA-AM) on the LPS-plus-trypsin-induced ERK/p38 MAPK phosphorylation and MCP-1 synthesis. EA. hy926 cells (1 × 10^6^/mL) were pretreated with BAPTA-AM (50 nM) for 30 min, after which they were stimulated with LPS (2 μg/mL) for 24 h and with trypsin (5 μg/mL) for 10 min. Control cells were treated with 0.1% DMSO. **a** Representative data of the p-ERK, ERK, p38, pp38 and MCP-1 protein levels, and β-actin was used as the loading control. **b** Normalization of the p-ERK and total ERK levels. **c** Normalization of the p-p38 and total p38 levels. **d** Normalization of the MCP-1 and total β-actin levels. Bar graphs represent means ± SEM from three independent experiments. **p* < 0.05 compared with the control group; #p < 0.05 compared with the LPS-plus-trypsin treatment group. (TIFF 4147 kb)

## Abbreviations

DMEM: Dulbecco’s Modified Eagle’s Medium
ECs: Endothelial cells
ERK: Extracellular signal-regulated kinase
FBS: Fetal bovine serum
GAPDH: Glyceraldehyde 3-phosphate dehydrogenase
JNK: c-Jun N-terminal kinase
LPS: Lipopolysaccharides
MAPK: Mitogen-activated protein kinase
MCP-1: Monocyte chemoattractant protein-1
PAR-2: Protease-activated receptor-2
PCR: Polymerase chain reaction
SDS: Sodium dodecyl sulfate
SEMs: Standard errors of the means
TBS: Tris-buffered saline
