# Supplementary figures and images for: Lipopolysaccharide pretreatment increases protease-activated receptor-2 expression and monocyte chemoattractant protein-1 secretion in vascular endothelial cells
Source: J Biomed Sci. 2017 Nov 15;24:85. doi: 10.1186/s12929-017-0393-1 (PMC5688698; doi:10.1186/s12929-017-0393-1)

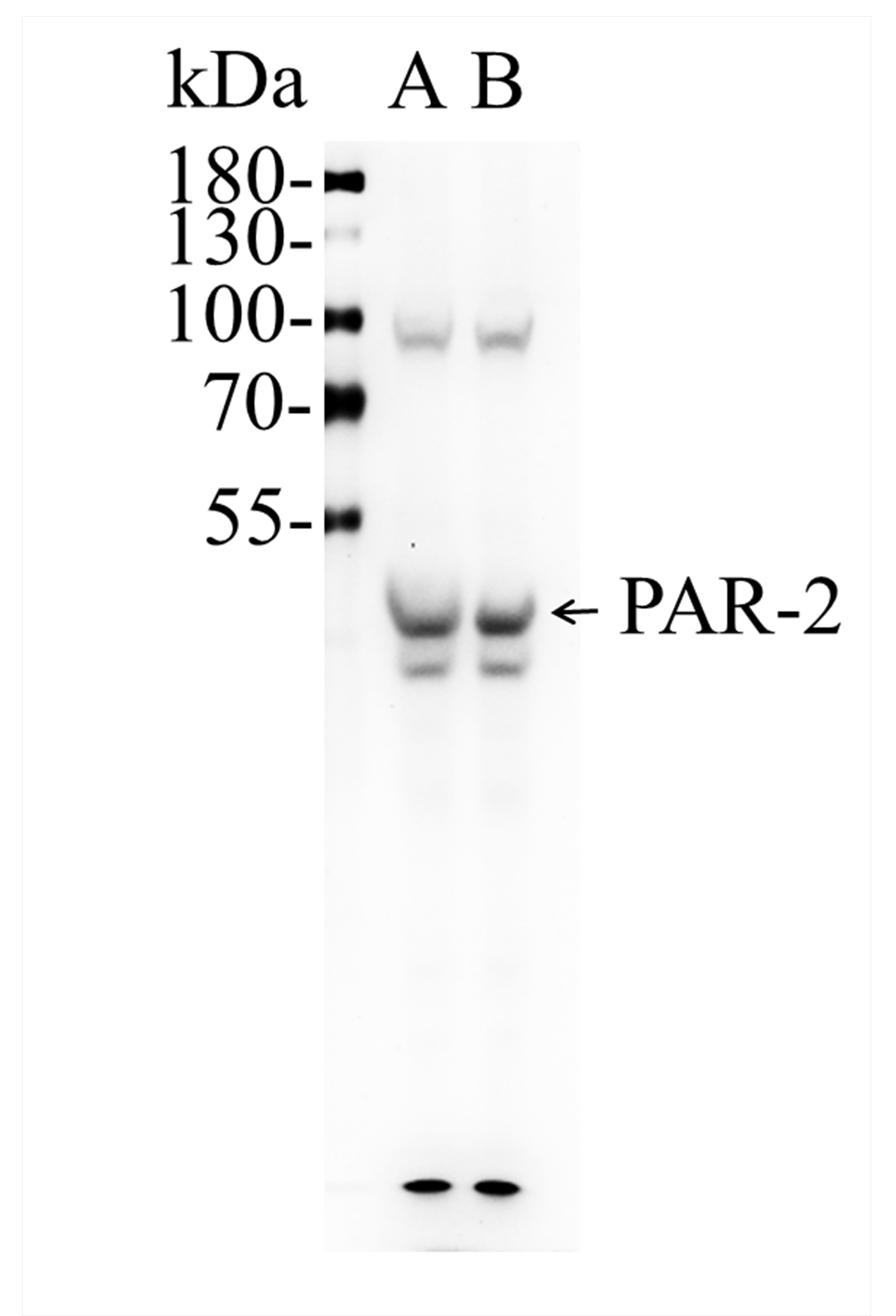

Supplement: Additional file 1: Figure S1. — Western blot – Anti-PAR-2 antibody. Lane A: Control cell lysate at 10 μg. Lane B: PAR-2 agonist (AC 55541, 10 μM)-treated cell lysate at 10 μg. Predicted band size: 43~55 kDa. (TIFF 823 kb) [file 12929_2017_393_MOESM1_ESM.tif]

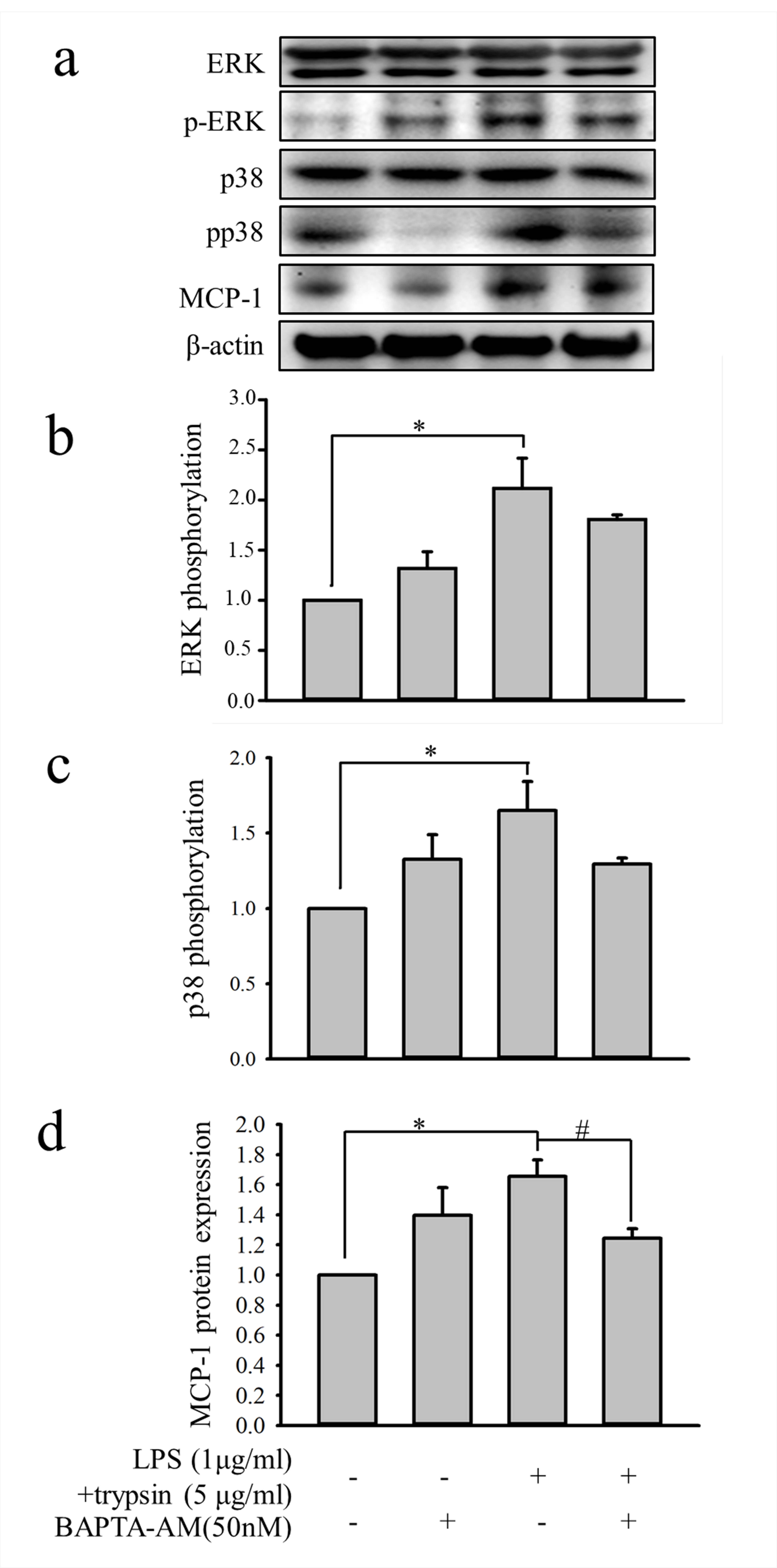

Supplement: Additional file 2: Figure S2. — The effect of calcium chelator (BAPTA-AM) on the LPS-plus-trypsin-induced ERK/p38 MAPK phosphorylation and MCP-1 synthesis. EA. hy926 cells (1 × 10^6^/mL) were pretreated with BAPTA-AM (50 nM) for 30 min, after which they were stimulated with LPS (2 μg/mL) for 24 h and with trypsin (5 μg/mL) for 10 min. Control cells were treated with 0.1% DMSO. a Representative data of the p-ERK, ERK, p38, pp38 and MCP-1 protein levels, and β-actin was used as the loading control. b Normalization of the p-ERK and total ERK levels. c Normalization of the p-p38 and total p38 levels. d Normalization of the MCP-1 and total β-actin levels. Bar graphs represent means ± SEM from three independent experiments. *p < 0.05 compared with the control group; #p < 0.05 compared with the LPS-plus-trypsin treatment group. (TIFF 4147 kb) [file 12929_2017_393_MOESM2_ESM.tif]
